# Lower limb injuries and their association with anthropometrics - an observational study of 381 German elite youth football players

**DOI:** 10.1186/s13102-025-01190-7

**Published:** 2025-06-04

**Authors:** Sebastian Viktor Waldemar Schulz, Lynn Matits, Eric Schwarz, Achim Jerg, Moritz Otte, Patrick Wiedemann, Daniel Alexander Bizjak, Johannes Kirsten, Alexander-Stephan Henze

**Affiliations:** 1 Sports and Rehabilitation Medicine, University Hospital Ulm, Ulm, Germany; 2 Faculty of Sports and Health Sciences, Munich Technical University, Munich, Germany; 3 Institute for Sports Science, Wuppertal University, Wuppertal, Germany; 4 Department of Sports Science, Humanities Section, Konstanz University, Konstanz, Germany; 5 Clinical & Biological Psychology, Institute of Psychology and Education, Ulm University, Ulm, Germany

**Keywords:** Soccer, Athletic injuries, Epidemiology, Genu varum, Primary prevention

## Abstract

**Background:**

Youth football players are at high risk of lower limb injuries, which can affect performance and long-term career development. However, epidemiological data on injury patterns in German elite youth football remain limited. This study aims to (1) provide an overview of lower limb injuries in German elite youth football academies and (2) investigate the association between anthropometric factors, particularly genu varum, and injury risk.

**Methods:**

This cross-sectional study included 381 healthy male football players (aged 10–23 years) from two German elite youth academies between 2021 and 2023. Injury history from the most recent full league season, including pre-season and regular season matches, was recorded. Anthropometric assessments included body composition and intercondylar distance of the knee (ICD) to evaluate genu varum.

**Results:**

A total of 140 lower limb injuries were documented, resulting in a cumulative injury frequency of 0.39 and an incidence of 1.2 injuries per 1,000 h. Most injuries were muscle-tendon injuries in the thigh (36.4%) and ligament injuries in the ankle (35.0%). Injury *hot spots* included lateral ligament tears in the ankle (30.0%) and muscle injuries within a triangle of the hamstrings (12.9%), quadriceps (10.7%), and adductors (11.4%) in the thigh. Injury frequency significantly increased from age 13 (*p* =.013; OR = 2.29), particularly in the thigh (*p* =.027; OR = 3.28). Regression analysis revealed a significant correlation between age (*p* =.038; coefficient of variation (COV) = -0.07) and ICD (*p* =.003; COV = -0.01) on the number of injuries, suggesting a potential link between genu varum and increased injury risk.

**Conclusion:**

This study provides the first structured overview of lower limb injuries in two German elite youth football academies and their association with anthropometric data. The findings highlight age-related injury patterns and suggest a potential link between genu varum and injury risk, underlining the need for targeted injury prevention strategies. Establishing a systematic, long-term injury surveillance system in youth football is essential for developing evidence-based interventions to reduce injury risk and support player development.

## Introduction

In the 2020/2021 season, players in German professional football (soccer) experienced an average of 2.1 injuries per season—significantly higher than in other contact sports such as basketball or ice hockey [1]. A similar trend is observed in international youth football, where elite male players sustain an average of 0.4 to 2.2 injuries per season. Approximately one in three players suffers at least one injury annually, with the majority involving the lower extremities [2, 3]. Injury rates in elite youth football (6.19 injuries per 1,000 h) exceed those in amateur youth football (4.77 injuries per 1,000 h), highlighting the greater physical demands at higher competition levels [2, 4]. Furthermore, he training-related injury rate for youth players is up to 4.9 times higher than for adults, emphasizing their heightened vulnerability [3].

Evidence also suggests that youth football players are at increased risk of injuries due to the immaturity of their musculoskeletal systems [5]. Injury incidence further varies based on factors such as age, competitive level, and regional playing conditions, necessitating population-specific research to develop effective prevention strategies [6]. Despite this need, there is a lack of up-to-date epidemiological data on injury incidence in German elite youth football, with existing data being over a decade old [7–9]. Establishing robust national baseline data is crucial to reducing bias in analyses of injury patterns and risk factors in highly selective elite youth populations.

The long-term goal should be to systematically identify injury patterns and associated risk factors, enabling the development of targeted prevention strategies to reduce injury burden, support player development, and optimize performance in German elite youth football [9]. Injury awareness is also vital for stakeholders to ensure that young talents remain engaged in structured training programs rather than being sidelined by injuries, recurrent trauma, or premature dropout [7, 10, 11]. Beyond performance setbacks, injuries at a young age can lead to long-term health consequences that persist into adulthood [12].

While it is impossible to eliminate all injuries, reducing their frequency and severity remains a shared priority. This study aims (i) to provide total career and most recent season lower limb injury data for healthy male players from two elite German youth football academies, and (ii) to investigate the association between lower limb injury risk and anthropometrics, particularly genu varum.

## Materials and methods

### Study design and population

This observational, cross-sectional study included 381 male football players from two German elite youth academies associated with professional men’s teams currently playing in the first and second national division. In 2022, the German football association (Deutscher Fußballbund, DFB) reached over 50 official elite youth football academies, which provided talented young players with systematic athletic and personal development under professional conditions, aiming to prepare them for potential professional careers [13]. Data for this study was collected during the annual pre-competition medical assessments (PCMAs) conducted in the pre-season periods of 2021, 2022, and 2023. The study adhered to the Consensus Statement on injury definitions and data collection procedures in football (soccer) injury studies [14] and followed the *Strengthening the Reporting of Observational Studies in Epidemiology* (STROBE) guidelines and their extension for Sport Injury and Illness Surveillance (STROBE-IIS) [15].

In both academies, training sessions for all age groups followed a standardized structure defined by the academy’s long-term development model. In general, U12 players trained three times per week (60–75 min per session), focusing on coordination, basic technical skills, and playful movement tasks. U13-U15 players trained four times per week (75–90 min), including structured strength and endurance components, football-specific drills, and basic tactical content. U16 and older players trained five times per week (90–105 min), with distinct sessions focusing on high-intensity aerobic work, strength/power training (2x/week), and tactical/technical integration. Weekly match frequency ranged from one official league match per week (U12-U15) to one to two matches plus friendlies or international matches in older age groups (U16-U23). All teams implemented injury prevention programs (e.g., FIFA 11 + or modified versions) integrated into warm-up routines at least twice per week. These session formats are consistent with those reported in elite youth football settings [6, 16].

Inclusion criteria required players to be aged 10–23 years, to have been with one of the clubs for at least one year, and to be free from chronic diseases such as diabetes mellitus, psoriasis, pancreatitis, or Chronic Obstructive Pulmonary Disease. For players with more than one year of club participation, only data from their most recent PCMA were analyzed. Exclusion criteria included withdrawal of Informed consent by the player, parent, or legal guardian. International training and match participation were not documented.

The study complied with the latest version of the Helsinki Declaration and was approved by the ethics committee of the University of Ulm (No. 371/23). Informed verbal consent was obtained from all participating players after they received age-appropriate information about the study’s aims, procedures, and their rights. Additionally, written informed consent was obtained from at least one parent or legal guardian prior to participation. Participant characteristics are presented in Table 1.

**Table 1:** Participant characteristics and incidence of lower limb injuries in elite German youth football players of the two academies.

Age group	Total players	Age	Body height	Body mass	BMI	FFM	FFM/ height	PBF	ICD	ICD/ height	Incidence/ 1,000 h	Playing level
	[n]	[years]	[cm]	[kg]	[kg/m^2^]	[kg]	[kg/m^2^]	[%]	[cm]	[*10^^−2^]		[national division]
U12	44	10.8 (0.4)	151.2 (8.9)	39.7 (8.1)	17.2 (1.8)	34.8 (7.4)	15.1 (1.5)	12.2 (4.4)	0.7 (0.9)	0.5 (0.6)	1.3	6th
U13	36	12.6 (0.5)	157.7 (7.4)	43.6 (7.0)	17.5 (2.1)	38.5 (6.0)	15.4 (1.5)	11.3 (6.3)	1.0 (1.3)	0.6 (0.8)	1.0	4th to 5th
U14	41	13.5 (0.5)	164.8 (9.2)	50.9 (10.9)	18.6 (2.5)	45.3 (9.1)	16.5 (1.9)	10.6 (4.5)	0.9 (1.2)	0.6 (0.7)	0.6	2nd to 3rd
U15	44	14.4 (0.5)	170.8 (7.8)	58.6 (10.1)	20.0 (2.3)	52.4 (8.4)	17.9 (1.7)	10.3 (4.6)	1.5 (1.2)	0.9 (0.7)	1.3	2nd to 3rd
U16	57	15.6 (0.5)	175.3 (7.4)	63.9 (7.8)	20.8 (2.0)	57.5 (7.1)	18.6 (1.3)	10.0 (4.4)	2.5 (1.9)	1.4 (1.1)	2.3	2nd to 3rd
U17	55	16.7 (0.5)	178.8 (6.1)	68.8 (7.3)	21.5 (1.6)	62.0 (6.7)	19.4 (1.4)	9.6 (3.8)	3.2 (2.3)	1.8 (1.3)	1.0	1st to 2nd
U19	74	17.5 (0.5)	179.6 (7.5)	71.8 (9.3)	22.2 (1.8)	65.2 (8.9)	20.1 (1.7)	9.2 (2.8)	2.8 (2.0)	1.5 (1.1)	1.6	1st to 2nd
U23	30	20.8 (1.6)	182.2 (6.0)	75.7 (5.2)	22.8 (1.4)	68.2 (4.7)	20.5 (1.0)	9.9 (3.5)	4.7 (1.6)	2.6 (0.8)	0.6	1st to 3rd

BMI = body mass index; FFM = fat-free mass; PBF = percent body fat; ICD = intercondylar distance. Note: data except total players, incidence and playing level is presented as mean and standard deviation.

### Data collection

The annual PCMA was conducted each summer before the start of the regular football season by the medical staff of the Sports and Rehabilitation Medicine department at the University Hospital Ulm, Germany. Once eligibility for study was confirmed, participants underwent additional assessments of anthropometry and injury status.

### Anthropometrics

Body composition was analyzed using bioelectrical impedance (BIA) technology (InBody 770, Biospace Korea, Seoul, Korea) to measure body mass [kg], fat-free mass (FFM) [kg/m²], and body fat percentage (BFA) [%] while unclothed except for underwear based on Kyle and colleagues with players having neither eaten nor drunk anything in the previous 1.5 h [17]. Body height was measured without shoes using a portable stadiometer with 0.1 cm precision (seca213, Hamburg, Germany).

The intercondylar distance (ICD), defined as the distance between the medial edges of the condyles, was measured with a digital caliper (Qfun, Shenzhen, China) with 0.01 mm accuracy. Measurements were taken in a standing position with the medial malleoli touching and the hips and knees fully extended [18]. ICD values were normalized to body height to account for individual stages of maturation. If no distance was present between the knee condyles, a value of zero was recorded [19].

All anthropometric measurements were performed by a single sports scientist with extensive experience in this field.

### Injury surveillance

The questionnaire of injury report adhered to the *Fédération Internationale de Football Association* (FIFA) *Medical Assessment and Research Center* (F-MARC) consensus on definitions and data collection procedures for football injury studies. It utilized translated versions of the forms developed by Fuller et al. [14]. The study recorded all traumatic musculoskeletal injuries, with a specific focus on lower limb regions (hip, thigh, knee, ankle, and foot) and injury types such as joint/ligament injuries, muscle/tendon injuries, and fractures. Details about the mechanisms of trauma were not captured.

Participants completed the questionnaire of injury report form by Fuller et al. [14] in collaboration with their parents, providing information on injuries sustained during their football careers and during the most recent full competitive season (usually one year), including pre-season and regular season matches. The competitive season was defined as the most recent full league season prior to data collection at the PCMA. The re-injury rate is defined as the recurrence of an injury at the same anatomical location within a single competitive season. Completed questionnaires of injury report form by Fuller et al. [14] were subsequently reviewed and verified by medical staff, including sports scientists and physicians from the Sports and Rehabilitation Medicine department at the University Hospital Ulm.

### Statistical analysis

Data were analyzed R (version 4.4.1, The R Foundation of Statistical Analysis) [20]. For descriptive analysis, mean and standard deviation were applied. Absolute and relative frequencies were used to compare injury locations and subtypes. Injury incidence was calculated as the number of injuries per 1,000 h of exposure during football.

Robust logistic regression models (R-package: robustbase [21]) were used to assess differences in injury risk and subtype probabilities during the most recent full competitive season across age groups. Age group was included as a contrast-coded predictor (≤ 12 vs. >12 years; 13–15 vs. >16 years), along with fat-free mass and the ICD/height ratio. Body fat was initially included but removed due to high multicollinearity (Variance Inflation Factor > 10). Multicollinearity diagnostics for all remaining predictors indicated acceptable levels.

Robust linear regression models (R-package: MASS [22]) were used to investigate the associations between intercondylar distance (ICD) relative to body height, fat-free mass (FFM), age, and the absolute number of injuries. Due to multicollinearity between FFM and body fat (*r* =–.99; variance inflation factor > 10), only FFM was retained as a predictor.

Effect sizes were estimated using partial eta squared (η²partial), which quantifies the proportion of explained variance attributable to each predictor while controlling for other variables. Effect sizes were interpreted according to Cohen’s guidelines [23], with values of η²partial ≥ 0.01 considered small, ≥ 0.06 medium, and ≥ 0.14 large. The significance level was set at *p* <.05.

## Results

During the study period, 381 of 427 players from two German elite football academies were included. The dropout reasons were missing data (10 players, 2,4%), club change within one competitive season (20 players, 4,7%), discontinued football participation (5 players, 1,2%), other reasons (11 players, 2,5%). An average of 48 (min-max: 30–74) players were recruited for each age group. The total cumulative football exposure of all players was 45,000 h, consisting of 39,240 h of training and 5,760 h of match play.

### Lower limb injuries from the most recent full competitive season

Of the 381 players, 111 (29.1%) reported at least one lower limb injury during most recent full competitive season, while 270 players (70.9%) remained injury-free. Among the injured players, 88 (79.3%) sustained a single injury, 17 (15.3%) experienced two injuries, and six (5.4%) reported three injuries. The overall re-injury rate was 21%. 22 players indicated that they had sustained at least one injury prior to the one-year competitive season.

**Fig. 1:**
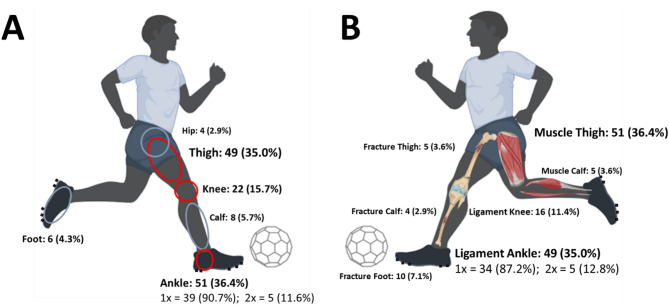
Overview of lower limb injuries (*n* = 140) from the most recent full competitive season in 381 male players from two German elite youth football academies, categorized by affected body region (**A**) and injury subtype (**B**). The first bold line shows the total number of injuries per region or injury subtype (n) and their relative proportion of all 140 injuries (%). The second line indicates the frequency of the specified injury region or subtype (1–2x) per player, including the number of cases per region or injury subtype (n) and the relative frequency within that region or subtype of injury (%).

A total of 140 lower limb injuries were documented, resulting in a cumulative injury frequency of 0.39. The most commonly affected body regions (see Fig. 1A) were the ankle (calf, ankle, and foot; 65 injuries, 46.4%), thigh (hip and thigh; 53 injuries, 37.9%), and knee (22 injuries, 15.7%). The most frequent injury subtypes (see Fig. 1B) were ligament injuries (65; 46.4%), followed by muscle-tendon injuries (56; 40.0%) and bone injuries (19; 13.6%).

Table 2 provides a detailed breakdown of lower limb injuries sustained by players throughout their football careers and during the one-year competitive season. The most frequent ligament injury subtype involved the lateral ankle ligaments, comprising 67.4% of all ligament injuries during players’ careers and 64.6% in the one-year competitive season. These were followed by anterior cruciate ligament (ACL) tears and knee meniscus injuries. The hamstring, quadriceps, and adductor triangle were the most commonly affected muscle-tendon groups in the thigh, accounting for 89.4% of all muscle-tendon injuries across players’ careers and 87.5% during the one-year competitive season. Bone fractures predominantly occurred in the metatarsus of the foot.

In summary, the three most common injury subtypes during the most recent full competitive season were: (1) Ligament injuries of the ankle (30.0% of all injuries), (2) Muscle injuries affecting the three primary thigh muscle groups (35.0% of all injuries), and (3) Foot fractures (5.7% of all injuries).

**Table 2:** Football career injuries and most recent full competitive season injuries of the lower limb with subtypes ligament, muscle-tendon, and bone in 381 male players from two German elite youth football academies.

Ligament injuries		Football career injuries			Previous competitive season injuries	
		Subtype injuries	All injuries		Subtype injuries	All injuries
	*n*	*n* = 89	*n* = 196	*n*	*n* = 65	*n* = 140
Ankle lateral ligaments	60	67.4%	30.6%	42	64.6%	30.0%
Ankle Syndesmosis	8	9.0%	4.1%	8	12.3%	5.7%
Ankle medial ligament	2	2.2%	1.0%	2	3.1%	1.4%
Knee anterior cruciate ligament tear	7	7.9%	3.6%	4	6.2%	2.9%
Knee meniscus	4	4.5%	2.0%	4	6.2%	2.9%
Knee lateral ligament	3	3.4%	1.5%	2	3.1%	1.4%
Knee medial ligament	3	3.4%	1.5%	2	3.1%	1.4%
Knee posterior cruciate ligament tear	1	1.1%	0.5%	0	0.0%	0.0%
Knee patella dislocation	1	1.1%	0.5%	1	1.5%	0.7%
**Muscle-tendon injuries**	**n**	*n* = 76	**196**	**n**	*n* = 56	*n* = 140
Thigh Hamstring	25	32.9%	12.8%	18	32.1%	12.9%
Thigh Quadriceps	22	28.9%	11.2%	15	26.8%	10.7%
Thigh Adductor	21	27.6%	10.7%	16	28.6%	11.4%
Calf lifters	6	7.9%	3.1%	5	8.9%	3.6%
Hip flexors	2	2.6%	1.0%	2	3.6%	1.4%
**Bone injuries**	**n**	*n* = 31	**196**	**n**	*n* = 19	*n* = 140
Metatarsal fracture	13	41.9%	6.6%	8	42.1%	5.7%
Ankle fracture	8	25.8%	4.1%	6	31.6%	4.3%
Big toe fracture	4	12.9%	2.0%	2	10.5%	1.4%
Femur growth plate splitting	1	3.2%	0.5%	1	5.3%	0.7%
Patella fracture	1	3.2%	0.5%	1	5.3%	0.7%
Physeal injury	1	3.2%	0.5%	1	5.3%	0.7%

### Injuries per age group

**Fig. 2:**
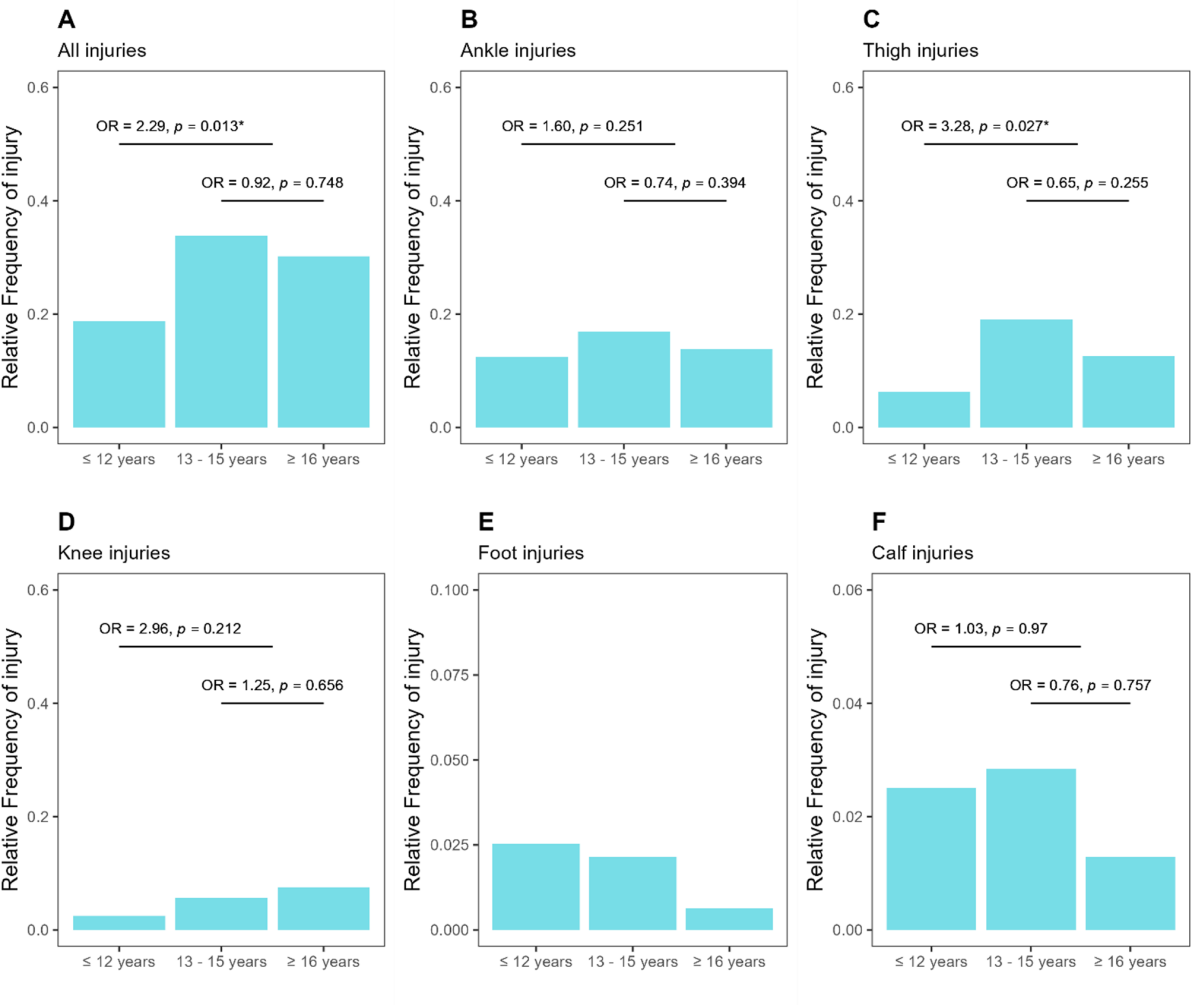
Relative frequency of most recent full competitive season injuries in 381 male players from two German elite youth football academies per age group in specific body regions [%]: **A** = all injuries; **B** = ankle injuries; **C** = thigh injuries; **D** = knee injuries; E = foot injuries; F = calf injuries. Significance set at *p*-value of * *p* ≤.050. OR = odds ratio.

Injury frequency data from the most recent full competitive season was categorized into three age groups: under 12 years, 13–15 years, and over 16 years, to account for growth and maturation factors [24]. Figure 2 illustrates injury frequencies across all categories (A) and specific body regions (B–F). From age 13 onward, injury frequency increased significantly, with the thigh being the most frequently affected region. While ankle and calf injuries also showed upward trends, these increases were not statistically significant. Knee injuries exhibited a steady rise with age, whereas foot injuries followed a declining trend.

**Fig. 3:**
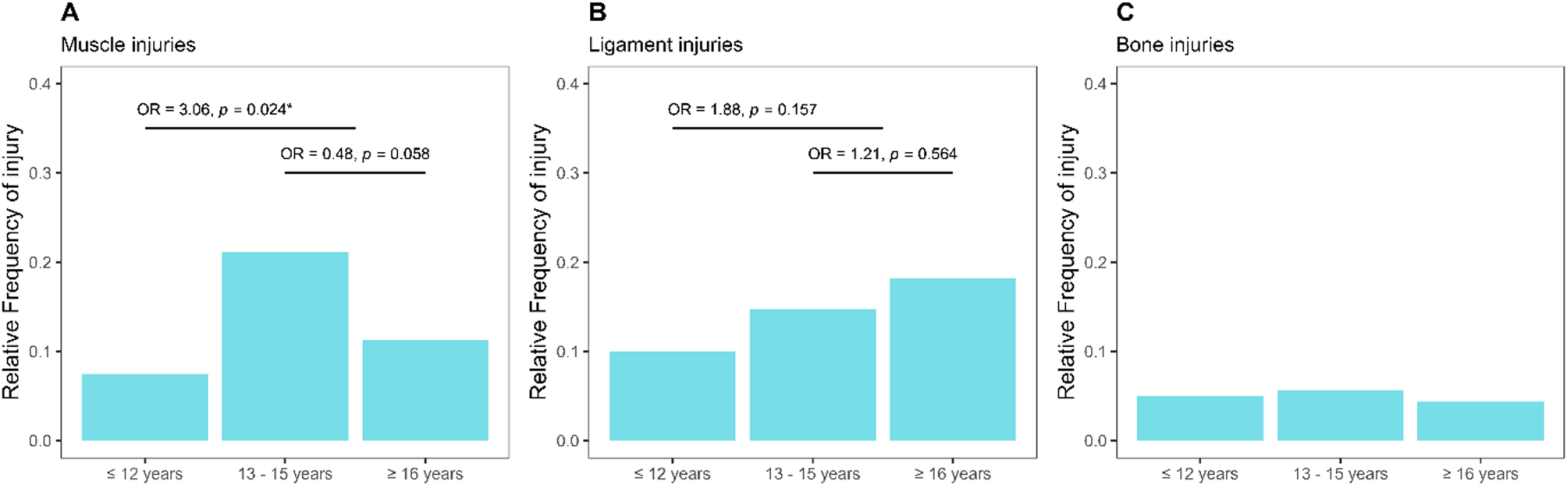
Relative subtypes of most recent full competitive season injuries in 381 male players from two German elite youth football academies per age group [%]: **A** = muscle-tendon; **B** = ligament; **C** = bone. Significance set at p-value of * *p* ≤.050. OR = odds ratio.

The subtype analysis (see Fig. 3) demonstrates a significant increase in muscle injuries in the 13–15 age group compared to younger players. However, the muscle injury rate declines in players over 16 years old. A similar pattern is observed for bone injuries, though the small sample size precludes meaningful statistical analysis. Ligament injuries, by contrast, show a cumulative rise across all three age groups, peaking in the over-16 age group.

### Association of most recent full competitive season lower limb injuries and anthropometrics

Regression analysis of one year’s competitive season data reveals a small negative correlation between ICD and lower limb injuries, while age shows a small positive correlation (Table 3).

**Table 3:** Linear regression between number of lower limb injuries and ICD/body height adjusted for age and FFM.

Parameter	Coefficient	95%-CI	SE	t (357)	η²_partial_ [95%-CI]	*p*
ICD/body height	-0.07	[-0.02, -0.00]	0.03	-2.08	0.01 [0.00, 0.04]	**0.038** ^*^
FFM [%]	10.96	[-0.19, 23.85]	0.66	1.67	0.00 [0.00, 0.04]	0.095
Age [years]	0.03	[0.01, 0.06]	0.01	2.95	0.02 [0.00, 0.06]	**0.003** ^**^

ICD = Intercondylar distance; ffm = fat free mass; se = standard error; ci = confidence interval; df = degrees of freedom; *p <.05, **p <.01. Note: to avoid multicollinearity, body fat [%] was removed from the linear regression model: variance inflation factor (VIF) > 10; correlation: *r* = −.99, ***p <.001.

## Discussion

In this observational, cross-sectional study of 381 male soccer players from two elite German youth soccer academies, football career injuries and injuries during the most recent full competitive season were recorded using a standardized questionnaire of injury report form by Fuller et al. [14] during the annual PCMA. In addition, as part of this PCMA, completed questionnaires of injury report form by Fuller et al. [14] were reviewed by the treating physicians with the players and their parents to ensure that the data collected was as accurate as possible. Anthropometric data on body composition and a varus alignment of the leg axis (ICD), which is common in football, were also collected.

Our main findings showed that these elite male youth football players are particularly prone to ankle, thigh, and knee injuries in the one-year competitive season. In addition, we found a significant increase in the frequency of most recent full competitive season lower limb injuries in the 13–15 and ≥ 16 age groups compared to the ≤ 12 age group. Finally, regression analysis revealed a significant negative correlation between body height normalized ICD and injury frequency.

### Football career and most recent full competitive season injuries of the lower limb

Recent research has reported injury incidence rates in international elite youth football academies in the age groups U9-U21 ranging from 1.3 to 21.1 per 1,000 h of play [6], with an overall rate of 6.3 per 1,000 h among boys [24]. In Germany, for example, an incidence rate of 2.6 per 1,000 h of exercise was observed in an elite youth academy for players aged U12–U19 [8]. In contrast, our study reported an injury incidence rate of 1.2 per 1,000 h in the U12–U23 age group. Variations in the reported injury rates may stem from differences in age structures within youth academies across countries [25, 26]. Additional contextual factors contributing to these discrepancies include levels of professionalization in youth football and constitutional differences among players [27, 28]. Furthermore, variations in injury data across studies may be attributable to the employed definitions of injury terms and reporting guidelines [15].

Regarding lower limb injuries, the most affected body regions in our data were the ankle (36.4%), thigh (35.0%), and knee (15.7%), consistent with existing literature [24]. However, Wik [24] reported different percentages, with 25% for the thigh and 18% for the ankle, while knee injuries (17%) were comparable. Notably, in our study, the ankle was the most affected lower limb region, with nearly half of the players experiencing a foot or ankle injury during the one-year competitive season.

Ligament injuries were the most common subtype in our cohort, accounting 46.4% of most recent full competitive season injuries, followed by muscle-tendon injuries (40.0%) and bone injuries (13.6%). Previous research indicates muscle injuries as the most frequent type, comprising 27–37% of all injuries in elite youth football, while ligament injuries account for 15–19% [8], but both types of injuries are the most common [2]. Despite this, our findings align with Jones et al. [6], identifying ligament sprains as a primary injury type (28.4%).

Table 2 highlights that lateral ankle ligaments were the most prevalent anatomical structures injured, affecting 30% of players in elite youth academies. Muscle strains or tears in the thigh were the second most prevalent injury, forming a pattern: quadriceps (10.7%), adductors (11.4%), and hamstrings (12.9%) were almost equally impacted. Additional prevalent injuries included other ankle ligament injuries (syndesmosis, medial ligament) and bone fractures (metatarsal, ankle, and big toe).

Knee injuries, such as anterior and posterior cruciate ligament (ACL/PCL) tears, patellar dislocations, and meniscal or cartilage injuries, were less frequent but associated with the longest recovery periods [29, 30] These severe injuries, despite their lower frequency, significantly impact player development and team performance. Moreover, serious injuries in youth football academies remain prevalent, reported at 18% [6] and 19% [8]. While ACL tears are rare in our data, their associated time-loss and a high recurrence rate—exceeding 10% after ACL replacement in young athletes—underscore the importance of preventive measures to minimize these adverse events.

In contrast to other studies, our research reported a relatively high number of bone injuries. Wik [24] suggests that adolescents are more susceptible to bone injuries due to reduced bone mineralization. However, this does not fully explain why fractures were rare among adolescent football players in other studies. The variation in injury subtype distribution may result from our study’s focus on traumatic-structural injuries, excluding overuse injuries.

Our data on football career injuries offer additional insights into the recurrence of injuries at the same location. Notably, the most common injuries also tended to recur most frequently. For instance, some players sustained up to four injuries in the ankle and thigh, while hip and calf injuries were reported only once. Regarding injuries from the past year, the ankle was the only region to sustain repeated structural injuries, reported twice.

The primary risk factors for re-injury in professional football include previous injuries and inadequate rehabilitation [31]. In Scandinavia, re-injury rates of 22% and 30% have been reported [32]. However, re-injury rates in youth football are significantly lower, with rates of 9.6% in Brazilian [33], and 3% in English [11] and German [8] football academies. This lower rate in youth football may be attributed to reduced financial pressure on players and clubs, allowing medical staff to prioritize evidence-based decisions focused on long-term player development. Additionally, the low re-injury rates in European youth football may reflect the influence of highly trained coaches in professional youth academies, where individualized training programs and support are becoming increasingly important [8].

### Injury data by age group

The categorization of players into different age groups provides a valuable framework for examining whether injury patterns vary with age and forms the foundation for further research and preventive strategies. Several studies have identified a peak in injury severity and burden among players in the U14–U16 age groups [5, 30, 34, 35], However, there is also evidence suggesting that injury frequency increases with age [9, 11, 30, 36, 37].

This age-related rise in injury risk can be attributed to several factors. As players grow older, they often gain weight, and training sessions and matches become increasingly intense, physically demanding, and competitive. Additionally, older players are more likely to have experienced previous injuries, which substantially heightens the risk of re-injury [38].

Physiological changes during growth spurts—such as more fragile growth plates, altered tissue adaptations, and reduced bone density—are frequently cited as contributing factors to the elevated risk of serious injuries. These changes, occurring during periods of peak height velocity (PHV) and peak weight velocity (PWV), place additional physical stress on young athletes, leading to a greater injury burden during these critical developmental stages.

The pubertal growth spurt in boys typically begins between 10 and 12 years of age, peaking at a growth rate of 8–10 cm per year between 13 and 15 years [24]. During this time, body weight also increases significantly, with a peak weight velocity (PWV) of 9–11 kg per year [39, 40]. These rapid physiological changes result in considerable variations in height and weight within the same age group [41]. Elevated growth rates, including changes in height, leg length, and BMI, are associated with a heightened risk of injury in young football players. Notably, the phases of peak height velocity (PHV) and PWV correspond to an increased risk of serious injuries that often lead to substantial time loss.

Our findings (Fig. 3) reveal that muscle injuries are most prevalent in the 13–15 age group but decrease thereafter, while ligament injuries show a steady increase with age. In addition to growth-related physiological changes, the increased physical demands associated with the transition to full-field play, longer match durations, and unchanged recovery intervals from the age of 13 onward may contribute to the observed increase in muscle injuries. These contextual training factors should be considered when interpreting age-related injury trends [26]. Analysis of injury locations (Fig. 2) indicates that ankle and calf injuries are also common within this age range. However, only the thigh region exhibits a significant accumulation of injuries.

The type of injury appears closely tied to absolute physical maturity. For instance, similar physical stresses are more likely to result in avulsion injuries in less developed players [42, 43], while more mature players are prone to muscle, ligament, or tendon injuries [24]. This observation aligns with the theory that growth-related injuries follow a bottom-up pattern, depending on maturity status and age [26]. This pattern mirrors the typical distal-to-proximal progression of skeletal maturation [44].

Our data further support this bottom-up pattern, showing a reciprocal progression of knee and foot injuries (Fig. 3). Specifically, knee injuries become more frequent with age, while foot injuries tend to decrease.

### Association of lower limb injuries and anthropometrics

Another focus our study was investigating the association between lower limb injuries and anthropometric measurements, including body composition and varus alignment of the leg axis. To ensure practical applicability, the measurements were designed to be simple and cost-effective, enabling their implementation in youth football practice. While previous studies have predominantly focused on age, they have rarely examined fat-free mass (FFM) and ICD.

In terms of age, our data confirms a positive correlation with small effect size, between age and the number of injuries [9, 30, 37, 45], with the trend becoming apparent from age 13 onward. Expanding the analysis to include additional age groups appears justified, as injury severity and burden often peak in the U14–U16 age group [30, 35]. To our knowledge, no published studies have specifically examined the relationship between body composition and number of injuries. Our analyses found no evidence supporting an association between FFM and injury. However, this result may be subject to bias, as elite football players generally exhibit superior lean body mass and fat mass metrics compared to those in the general healthy or obese population [46].

We observed an increase in ICD among elite male players over the years [18, 19, 47]. In previous research, genu varum has been associated with higher risk of injury [48] and an increased risk of injury, including a 1.3- to 2.9-fold higher likelihood of developing knee osteoarthritis in adult professional football players [49]. However, the regression analyses in the present study revealed a negative association - with a small effect size between ICD and injury frequency in youth football players, indicating that greater ICD was associated with fewer injuries. This finding is novel and warrants further investigation. It is possible that players with genu varum may possess certain advantages in football-specific skills. Another potential avenue for research could involve examining the role of muscles responsible for knee control and ankle stabilization—such as the gluteus medius, quadratus lumborum, and ischiocrural muscle groups [48, 50] - in players with varying levels of ICD.

Sport-specific exercises like running, along with flexibility, balance, and strength training, could prevent injuries. Programs like FIFA 11 + and FUNBALL, reducing injury rates by up to 31%, focus on muscle strength and control [51–61]. Factors such as equipment, weather, motivation, and communication among medical teams, players, and coaches are also essential [62, 63]. The return-to-sport framework guides decisions using risk assessment models, aiding clinical practice and research [63]. However, standardizing return-to-sport outcomes and identifying key prognostic factors require further study [59].

### Limitations

The main limitation of this observational study is a “recall bias” in the reporting of injuries [64]. To minimize this bias, the parents assisted in completing the questionnaire of injury report form by Fuller et al. [14]. Based on the completed questionnaire of injury report form by Fuller et al. [14], the medical history was then carefully reviewed by the study physicians together with the parents. To reduce inter-rater variability, an orthopedic surgeon and sports medicine specialist reviewed questionnaires of injury report form by Fuller et al. [14] with participants and parents, while performance diagnostics and anthropometric measurements were conducted by only one experienced sports scientist. Furthermore, this questionnaire did not collect information on the injury situation (e.g., direct/indirect contact or non-contact injury, severity of injury or associated time loss for training and competition).

Radiographic analysis is the gold standard for assessing lower limb length, axis, and angles, including the detection of genu varum [65]. However, its use is limited by the risks associated with radiation exposure [66]. The clinical ICD measurement method, while more susceptible to practical application errors, has proven to be an excellent alternative when performed by trained personnel [47, 67].

We documented zero if there was no space between the knee condyles, as football players showed a significantly higher mean ICD (1.5 cm, 95% CI [0.53, 2.46]) compared to non-football players. Nevertheless, this procedure may lead to bias in the interpreting of the injury data.

A limitation is the estimation of total exposure time. The 45,000 h were derived from standardized practice and game schedules and individual participation data. While team logs and coaching reports validated participation, exposure was not recorded on a per-player, per-session basis. Thus, estimates are approximate and may not reflect individual variation due to illness or absences. This approach, consistent with previous large-scale youth football studies, limits the precision of exposure-related injury rates.

## Conclusion

Elite youth football players face extensive training and competition demands, increasing their risk of injury. Injuries are critical as they can lead to exclusion from development programs, reducing the likelihood of advancing to professional football. Our data indicate that lower limb injury frequency rises significantly after age 12, with age-related differences in affected body regions and anatomical structures. Injury hot spots include the ankle, thigh, and knee. The role of intercondylar distance (ICD) in adolescent injuries warrants further exploration in future research.

Based on these findings, targeted prevention programs should be adapted to different age groups. For players under 12 years old, interventions should prioritize balance training and low-intensity muscle strengthening for the thigh. For U13-U15 players, neuromuscular training should be intensified to reduce injury risk in subsequent age categories.

Our study is the first to provide an up-to-date overview of lower limb injuries in two German elite youth football academies. The research aims to initiate long-term, systematic injury and illness surveillance in these youth football academies, incorporating anthropometric, physiological, psychological, personality, and performance data, supporting the development of future prevention strategies.

## Data Availability

The datasets used and/or analysed during the current study are available from the corresponding author on reasonable request.

## Declarations

### Authors' information

SS: sport scientist (PhD), athletic trainer and physiotherapist; ES: sport scientist and athletic trainer; DAB: biochemist and scientists (PD); ASH: orthopedic surgeon and sports medicine specialist; MO: sport scientist; PW: sport scientist; AJ: internal medicine physician and biologist; LM: psychologist and statistician; JK: internal medicine physician and sports medicine specialist.

## Abbreviations

ACL: Anterior cruciate ligament
BFA: Body fat percentage
CI: Confidence interval
COV: Coefficient of variation
DF: Degrees of freedom
DFB: Deutscher Fußballbund
FIFA: Fédération Internationale de Football Association
FFM: Fat-free mass
F-MARC: Medical Assessment and Research Center
ICD: Intercondylar distance of the knee
PCL: Posterior cruciate ligament
PCMA: Pre-competition medical assessments
PHV: Peak height velocity
PWV: Peak weight velocity
SE: Standard error
STROBE: Strengthening the Reporting of Observational Studies in Epidemiology
STROBE-IIS: Sport Injury and Illness Surveillance
VIF: variance inflation factor
